# miR-370-3p affects the progression of postmenopausal osteoporosis through targeting INO80

**DOI:** 10.1186/s41065-025-00502-8

**Published:** 2025-07-22

**Authors:** Zhen Yang, Yuqi Sheng, Xiangjie Liu, Meini Cen, Yong Xu

**Affiliations:** 1 Hepatic Department, The Affiliated Hospital of Hangzhou Normal University, Hangzhou, 310015 China; 2 School of Basic Medical Sciences, Zhejiang Chinese Medical University, Hangzhou, 310053 China; 3 Department of Orthopedics, The First Affiliated Hospital of Chengdu Medical College, Chengdu, 610500 China; 4 Department of Rehabilitation Medicine, The Affiliated Hospital of Youjiang Medical University for Nationalities, Baise, 533000 China; 5 Guangxi Key Laboratory for Preclinical and Translational Research on Bone and Joint Degenerative Diseases, Baise, 533000 China; 6 Department of Bone Disease and Sports Medicine, Hunan University of Medicine General Hospital, No.144 Jinxi South Road, Hecheng District, Huaihua City, 418000 Hunan China

**Keywords:** miR-370-3p, INO80, Postmenopausal osteoporosis

## Abstract

**Background:**

Postmenopausal osteoporosis (PMO) is acknowledged as a principal category of osteoporosis (OP). The aim of this study was to investigate the level of miR-370-3p in PMO patients and its predictive effect on osteoporosis in postmenopausal women, and to explore the molecular mechanism of miR-370-3p on PMO.

**Methods:**

The expression of miR-370-3p was assessed using RT-qPCR. Cell proliferation of MC3T3-E1 cells was evaluated through CCK-8 assays. Cell apoptosis was detected by flow cytometry. The direct interaction between miR-370-3p and INO80 was confirmed via dual-luciferase reporter assays.

**Results:**

The level of miR-370-3p was found to be upregulated in osteoporosis patients, and miR-370-3p played a significant role in regulating the proliferation, apoptosis and differentiation of osteoblasts. In addition, miR-370-3p targeted INO80 and affected the disease progression of PMO.

**Conclusions:**

miR-370-3p/INO80 may serve as a promising biomarker for both the diagnosis and therapeutic management of PMO.

**Supplementary Information:**

The online version contains supplementary material available at 10.1186/s41065-025-00502-8.

## Background

Osteoporosis (OP) is a condition characterized by a reduction in bone mass and degeneration of micro-architecture, which commonly increases the risk of bone fragility and fractures [1]. As the global population ages, the prevalence of osteoporosis is emerging as a critical public health, particularly affecting postmenopausal women and the elderly [2]. Postmenopausal osteoporosis (PMO) is acknowledged as a principal category of osteoporosis. Women who have reached menopause are more susceptible to developing osteoporosis because of various elements, including advancing age, estrogen deficits, and calcium inadequacies [3]. A range of therapeutic options is currently available for managing PMO, such as bisphosphonates, calcitonin, and certain other drugs. Nonetheless, these treatments are often accompanied by adverse gastrointestinal effects [4]. Accordingly, it is essential to further investigate the underlying mechanisms of PMO and to develop more effective and targeted therapeutic strategies.

Numerous microRNAs (miRNAs) play pivotal roles in regulating a wide array of pathophysiological processes, including cell proliferation and differentiation [5], autophagy [6], and inflammatory response [7]. It is estimated that miRNAs exert regulatory control over approximately 30% of human genes. To date, more than 2,500 miRNAs have been identified in humans [8]. A multitude of miRNAs exhibiting differential expression have been identified as associated with the molecular pathogenesis of OP, including miR-21-5p [9], miR-206 [10], miR-151a-3p [11], and others. Additionally, miRNAs get involved in the differentiation and function of bone cells, as well as in bone development and related diseases [12], including osteoarthritis [13], tendon injuries [14].

The role of miR-370 in various diseases has been reported. For instance, miR-370 has been identified as both a tumor suppressor and promoter across different cancer types [15, 16]. miR-370 serves as a non-invasive biomarker for the diagnosis of pediatric acute myeloid leukemia [17]. miR-370-3p has been recognized as a biomarker of sepsis-induced acute kidney injury [18]. Additionally, miR-370-3p plays a pivotal role in the development of adipocytes and the regulation of fatty acid metabolism [19]. Furthermore, the expression levels of miRNAs were determined and forecasted in the peripheral blood mononuclear cells (PBMCs) of mice with osteoporosis, with 13 miRNAs being markedly upregulated, including miR-370-3p [20]. Nonetheless, the clinical significance and underlying molecular mechanisms of miR-370-3p in patients with PMO remain elusive. Therefore, we hypothesize that miR-370-3p is abnormally expressed in patients with osteoporosis and may be involved in the disease progression of osteoporosis.

This study aimed to investigate the expression of miR-370-3p in PMO patients and the predictive effect of miR-370-3p on PMO, as well as to explore the molecular mechanism of miR-370-3p on PMO.

## Methods

### Subjects

Subjects for this study were recruited from The First Affiliated Hospital of Chengdu Medical College. A total of 129 postmenopausal females was enrolled in the study, comprising 64 postmenopausal females diagnosed with OP and an additional 65 postmenopausal females serving as controls without OP. Power analysis using G*power software confirmed that when *n* = 129, the calculated power value was greater than 0.8, indicating that the sample size was adequate. In addition, this study included another 20 postmenopausal non-OP individuals and 20 postmenopausal OP patients from the same period as independent cohorts. All patients were diagnosed according to the diagnostic criteria [21]. Participants had to meet the following criteria: (1) no history of smoking or alcohol use; (2) no history of chronic malignant diseases or genetic disorders; (3) no history of hormone replacement therapy. 5 ml of fasting blood was drawn from all subjects. After standing for 10 min, the samples were centrifuged for 10 min. The all samples were stored in ultra-low temperature freezers at -80℃ for future analysis. Within 7 days of serum extraction, the expression of miR-370-3p in serum was detected by RT-qPCR.

All subjects provided written informed consent. The study was approved by the Institutional Ethical Committee of The First Affiliated Hospital of Chengdu Medical College (No. CYFY18032023). All procedures performed in studies involving human participants were in accordance with the ethical standards of the institutional and/or national research committee and with the 1964 Helsinki Declaration and its later amendments or comparable ethical standards.

### Cell culture

The MC3T3-E1 cells (ATCC, Manassas, VA, USA) were used between passages 3 and 6. Cells were cultured in alpha-modified minimal essential medium (α-MEM; Gibco, Grand Island, NY, USA) with 10% fetal bovine serum (FBS, Gibco) and 1% penicillin/streptomycin, and incubated at a temperature of 37 °C at an incubator containing 5% CO_2_. MC3T3-E1 cells were plated at 2 × 10⁵ cells/well, the process of osteogenic differentiation was induced by a concentration of 50 mg/L of ascorbic acid and 10 mM/L of β-glycerophosphate, for 0, 7, and 15 days. Subsequently, the expression of miR-370-3p and INO80 was detected at each time point, and the expression of differentiation markers was detected 15 days after differentiation.

### Cell transfection

MC3T3-E1 cells at 70% confluence were transfected with 50 nM of each construct: miR-370-3p mimics (to enhance the expression of miR-370-3p) and miR-370-3p inhibitor (to suppress the expression of miR-370-3p) (GenePharma Co., Ltd., Shanghai, China). This process conducted by using Lipofectamine 2000 (Invitrogen, Carlsbad, California, USA) for 48 h. To inhibit the level of INO80, cells were transfected with siRNA targeting INO80 (si-INO80) (GenePharma Co., Ltd., Shanghai, China).

### RNA extraction and RT-qPCR

The total RNAs from serum and cells were extracted using TRIzol reagent (Invitrogen, Carlsbad, CA). cDNA was synthesized utilizing the RevertAid™ H Minus First Strand cDNA Synthesis Kit (Fermentas; Hanover, NH, USA). Reverse transcription quantitative polymerase chain reaction (RT-qPCR) was conducted employing the ABI 7500 Real-Time PCR System (Biosystems; Foster, CA, USA). Relative expression levels were calculated using the 2^−ΔΔCt^ method, with GAPDH and U6 genes serving as reference controls. The primer sequences are as follows:

miR-370-3p forward: 5’-TCGGCAGGGCCUGCUGGGGUGG-3’.

miR-370-3p reverse: 5’-CTCAACTGGTGTCGTGGA-3’.

INO80 forward: 5’- GCAAAGCCCCTTTATCTTCAGT − 3’.

INO80 reverse: 5’- CCAGAGGTTTCGCCAAGCAA − 3’.

GAPDH forward: 5’-ACCTGACCTGCCGTCTAGAAAA-3’.

GAPDH reverse: 5’-CCTGCTTCACCACCTTCTTGA-3’.

U6 forward: 5’- CGCAAGGATGACACGCAAAT-3’.

U6 reverse: 5’- GCAGGGTCCGAGGTATTC-3’.

### Cell counting kit (CCK-8) assay

The evaluation of cell viability was conducted using the CCK-8 assay. Cells viability was assessed at 24, 48, 72 h after transfection, then 10 mL of CCK-8 reagent (Dojindo, Japan) was added to each well containing MC3T3-E1 cells at a density of 5 × 10^3^ per well. Following a 2-hour incubation period, the absorbance at a wavelength of 450 nm was measured.

### Cell apoptosis assay

The cell apoptosis was detected using flow cytometry. An Annexin V-FITC Apoptosis Detection Kit (Keygen Biotechnology) was utilized for the quantitative assessment of apoptosis. Cells were plated at a density of 1 × 10^6^ cells per well in a 6-well plate. Following a 24-hour incubation period, the cells were harvested and gently washed with PBS. Subsequently, the cells were resuspended in 0.5 mL of PBS to achieve a cell concentration exceeding 1 × 10^5^ cells. Then, 5 µL of Annexin V-FITC solution and 1 µL of PI staining solution were added, and the mixture was incubated in the dark for an additional 15 min. Finally, the apoptosis rates were measured with a flow cytometer.

### Dual-Luciferase reporter assay

The 3’-untranslated region (3’-UTR) of either the mutant (MUT) or wild-type (WT) INO80 was inserted into dual luciferase reporter vectors (Promega, Madison, WI, USA). Subsequently, the WT or MUT INO80 plasmids, along with miR-370-3p mimic, inhibitor, or a negative control (miR NC), were co-transfected into cells. After a 48-hour incubation following transfection, luciferase activities were measured using the Dual-Luciferase Reporter Assay Kit.

### Western blot

Protein extraction was performed on the collected cells, followed by quantification of the total protein concentration using a BCA assay kit (Boster Biological Technology Co. Ltd). The extracted proteins were then mixed with a loading buffer and subjected to heat denaturation at 95 °C. Subsequently, 20 µg of protein per lane was loaded onto a 10% polyacrylamide gel (Boster Biological Technology Co. Ltd.) for electrophoretic separation. Following electrophoresis, the separated proteins were transferred onto a PVDF membrane, which was then incubated for 1 h at room temperature in 5% BSA to block non-specific binding sites. The membrane was incubated overnight at 4 °C with primary antibodies targeting ALP (ab307726, Abcam, Shanghai, China), OCN (ab133612, Abcam, Shanghai, China), RUNX2 (ab192256, Abcam, Shanghai, China), and β-actin (ab8226, Abcam, Shanghai, China). After three washings, the membrane was incubated with secondary antibodies for 1 h at room temperature. The membrane underwent another series of three 5-minute washes prior to detection using an ECL chemiluminescence reagent. Image J software was utilized to analyze the grayscale intensity of the target protein bands.

### Statistical analysis

The statistical analysis of all data was conducted using GraphPad Prism 9. 0 and SPSS 26.0 software. Differences between two groups were analyzed via the student’s *t*-test, while variances among multiple groups were analyzed through one-way analysis of variance (ANOVA), and the Turkey test method was used to correct the results of multiple comparisons. Data are presented as the mean ± standard deviation (SD). Receiver operating characteristic (ROC) curve was used to evaluate the diagnostic value of miR-370-3p in PMO patients. Multivariate regression analysis was used to explore the influencing factors of osteoporosis in postmenopausal women. *P* < 0.05 indicated the difference was statistically significant.

## Results

### Expression and clinical predictive effect of miR-370-3p in osteoporosis

The age and BMI of the control group and PMO patients did not show significant difference, but the lumbar spine bone mineral density (LS BMD), femoral neck bone mineral density (FN BMD), and total hip bone mineral density (TH BMD) in PMO patients were significantly lower compared to the control group. The average menopausal duration of patients in the osteoporosis group was longer than that of the non-osteoporosis group. However, despite this trend, there was no significant statistical difference. In addition, there was no significant difference in the calcium intake rate between the two groups of women (Table 1).

**Table 1:** Clinical data of the study population

Parameter	Control (*n* = 65)	OP patients (*n* = 64)	*P* value
Age (years)	52.62 ± 4.06	51.72 ± 3.91	0.204
BMI (kg/m^2^)	22.57 ± 3.22	23.07 ± 3.19	0.376
LS BMD (g/cm^3^)	0.94 ± 0.06	0.84 ± 0.05	<0.001
FN BMD (g/cm^3^)	0.74 ± 0.06	0.63 ± 0.05	<0.001
TH BMD (g/cm^3^)	0.77 ± 0.06	0.65 ± 0.05	<0.001
Menopausal duration	4.89 ± 2.55	5.55 ± 2.56	0.146
Calcium intake (yes/no)	37/28	29/25	0.218

Data are expressed as n or mean ± standard deviation. OP: osteoporosis, BMI: body mass index, LS BMD: lumbar spine bone mineral density, FN BMD: femoral neck bone mineral density, TH BMD: total hip bone mineral density

**Fig. 1:**
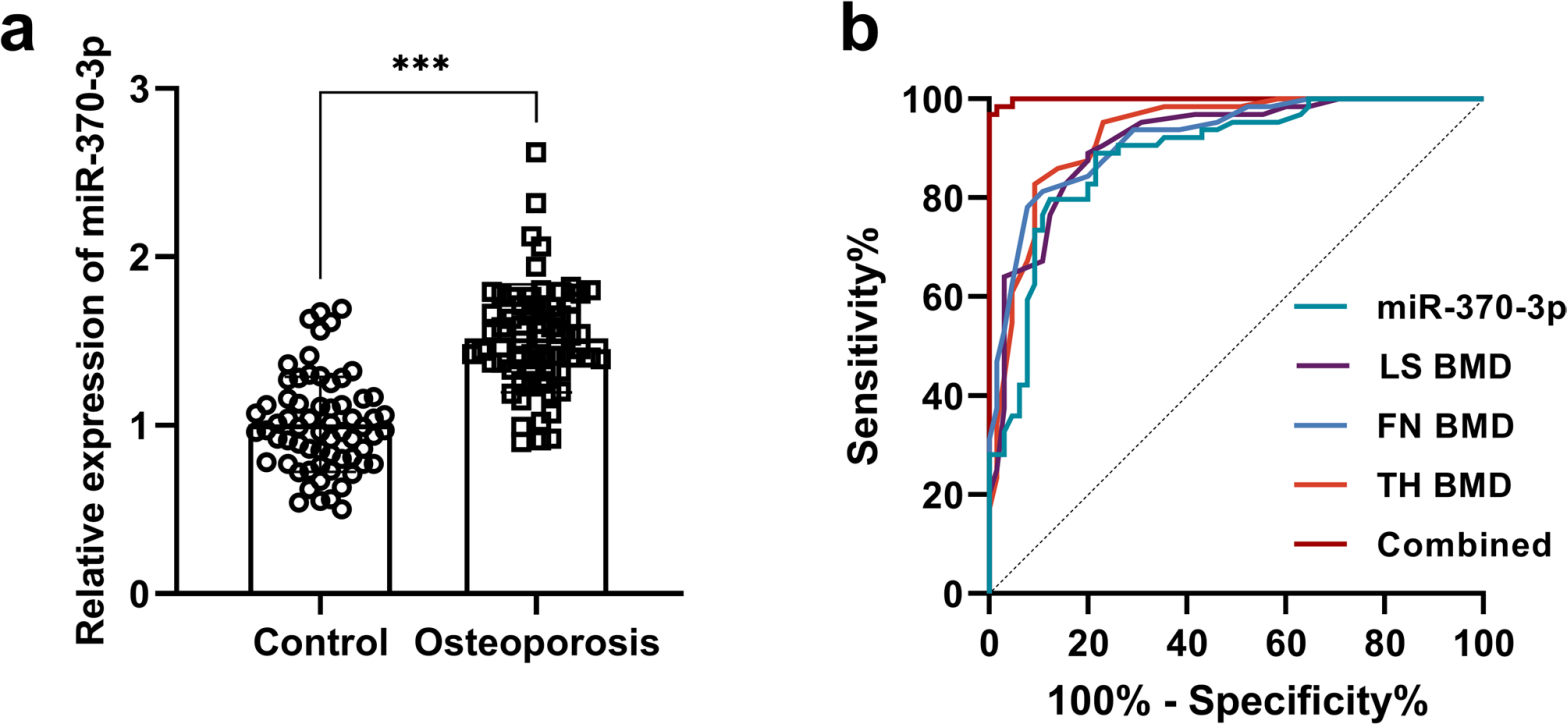
Expression and clinical predictive effect of miR-370-3p in osteoporosis. ^***^p < 0.001, ^**^p < 0.01, ^*^p < 0.05. Expression of miR-370-3p in control group (*n* = 65) and PMO patients (*n* = 64) (**a**). ROC curve of miR-370-3p and BMD in predicting osteoporosis in postmenopausal women (**b**)

The expression level of miR-370-3p in PMO patients was significantly higher than that in postmenopausal women without OP (Fig. 1a). In addition, in the independent validation cohort, miR-370-3p exhibited a significant upward trend (Supplementary Fig. 1). The ROC curve was conducted according to the levels of miR-370-3p in PMO patients and controls. The ROC curve yielded an AUC of 0.884, with a sensitivity at 79.69% and a specificity at 87.69%. In addition, LS BMD, FN BMD, and TH BMD also served as efficient indicators for diagnosing OP in postmenopausal females, and miR-370-3p showed higher diagnostic efficacy when combined with these indicators (Fig. 1b). In addition, the multivariate logistic regression analysis showed that after adjusting for other confounding factors, miR-370-3p remained an independent influencing factor for the occurrence of osteoporosis in postmenopausal women (Table 2). Consequently, serum miR-370-3p expression may serve as a promising biomarker for distinguishing between OP patients and those without OP.

**Table 2:** Multivariate logistic regression analysis of osteoporosis in postmenopausal women

Indicators	Odds Ratio	95%CI		*P* value
		low limit	up limit	
miR-370-3p	6.625	2.106	20.841	0.001
AGE	1.131	0.366	3.497	0.831
BMI	1.330	0.439	4.025	0.614
LS BMD	0.149	0.048	0.464	0.001
FN BMD	0.165	0.054	0.503	0.002
TH BMD	0.192	0.063	0.588	0.004
Menopausal duration	2.569	0.815	8.096	0.107
Calcium intake	0.536	0.177	1.628	0.271

BMI: body mass index, LS BMD: lumbar spine bone mineral density, FN BMD: femoral neck bone mineral density, TH BMD: total hip bone mineral density

**Fig. 2:**
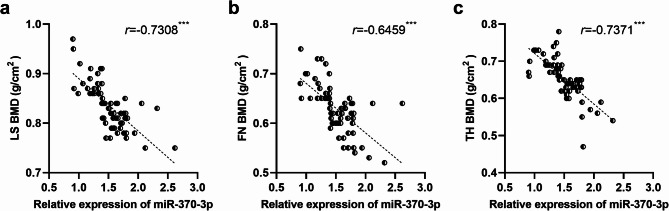
Correlation between miR-370-3p and BMD in patients. ^***^p < 0.001. The expression level of miR-370-3p was negatively correlated with LS BMD (**a**), FN BMD (**b**) and TH BMD (**c**) in PMO patients

Moreover, it was revealed that the expression levels of miR-370-3p were negatively correlated with the measurements of LS BMD, FN BMD, and TH BMD in patients with OP (Fig. 2a and c).

### Effects of miR-370-3p on osteoblasts

**Fig. 3:**
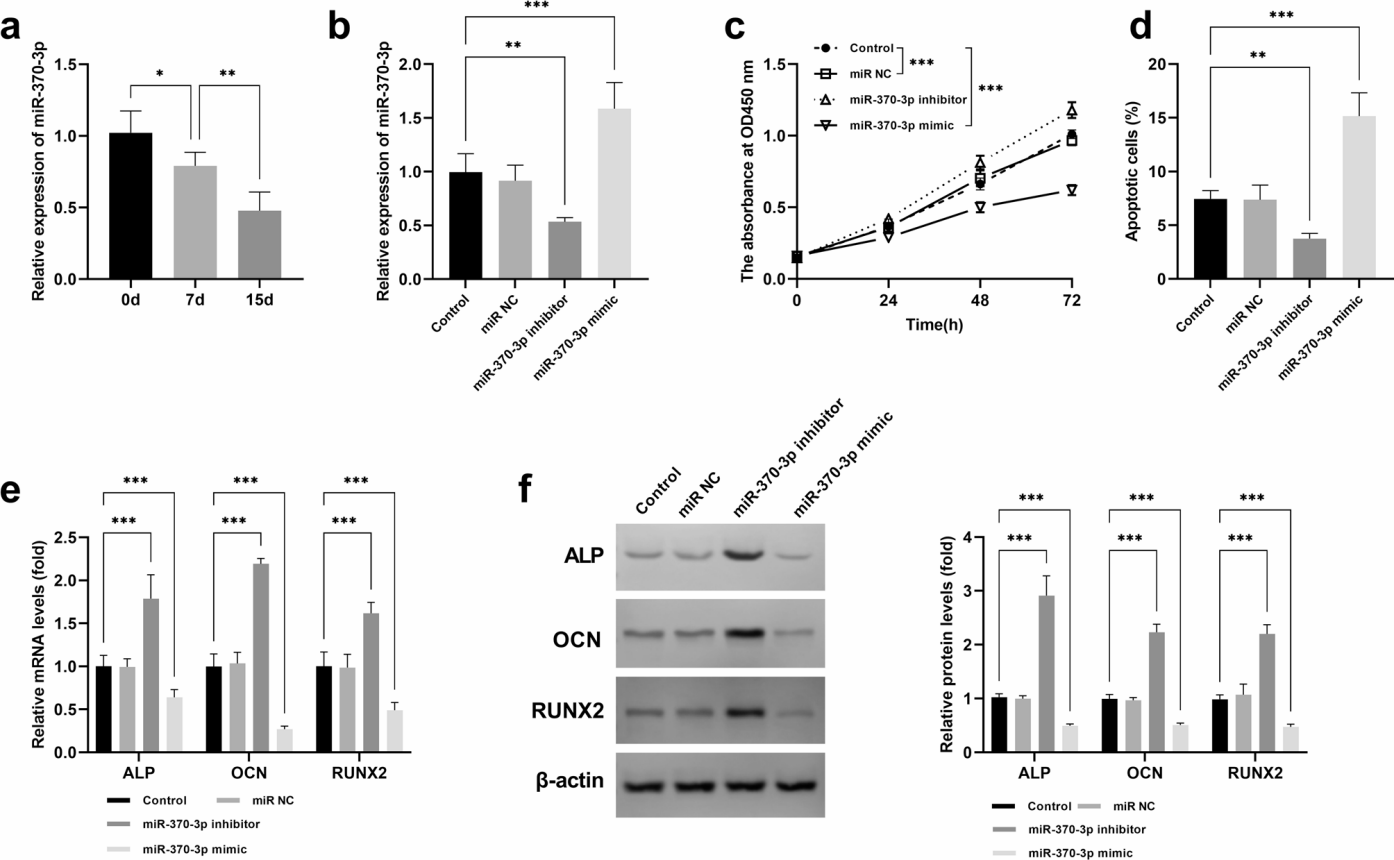
Effects of miR-370-3p on osteoblasts. ^***^p < 0.001, ^**^p < 0.01. Expression of miR-370-3p at different times of osteoblast differentiation (**a**). Expression level of miR-370-3p in MC3T3-E1 cells after transfection of miR-370-3p mimic and miR-370-3p inhibitor (**b**). Effect of miR-370-3p mimic and miR-370-3p inhibitor on cell proliferation (**c**) and apoptosis (**d**). Effect of miR-370-3p mimic and miR-370-3p inhibitor on the mRNA expression of osteoblast differentiation markers after 15 days of differentiation (**e**). Effect of miR-370-3p mimic and miR-370-3p inhibitor on the protein levels of osteoblast differentiation protein markers after 15 days of differentiation (**f**)

The level of miR-370-3p exhibited a gradual decline with the extension of cell differentiation time (Fig. 3a). In addition, as the differentiation time prolonged, the mRNA expression levels of osteoblast differentiation markers (ALP, OCN, RUNX2) also increased gradually, which verified the osteoblast differentiation and maturation (Supplementary Fig. 2). The effect of miR-370-3p on osteoblasts was studied by transfecting miR-370-3p mimic or miR-370-3p inhibitor into cells. Figure 3b showed that the expression of miR-370-3p was down-regulated after transfection with miR-370-3p inhibitor, and up-regulated after transfection with miR-370-3p mimic. In addition, the inhibition of miR-370-3p resulted in enhanced proliferation and a reduced apoptosis rate, whereas overexpression of miR-370-3p led to a decrease in osteoblast proliferation and an increase in apoptosis rate (Fig. 3c and d). In addition, miR-370-3p inhibition significantly increased the levels of ALP (1.79 ± 0.28-fold), OCN (2.19 ± 0.06-fold), RUNX2 (1.62 ± 0.13-fold); while the miR-370-3p mimic reduced the levels of ALP (0.64 ± 0.09-fold), OCN (0.27 ± 0.04-fold), and RUNX2 (0.49 ± 0.09-fold) (Fig. 3e). Moreover, when miR-370-3p was inhibited, the protein levels of ALP (2.91 ± 0.37-fold), OCN (2.23 ± 0.15-fold), and RUNX2 (2.20 ± 0.17-fold) were increased. In contrast, overexpression of miR-370-3p led to a decrease in the protein expression levels of ALP (0.49 ± 0.03-fold), OCN (0.51 ± 0.04-fold), and RUNX2 (0.47 ± 0.05-fold) (Fig. 3f).

### INO80 is one of the targets of miR-370-3p

**Fig. 4:**
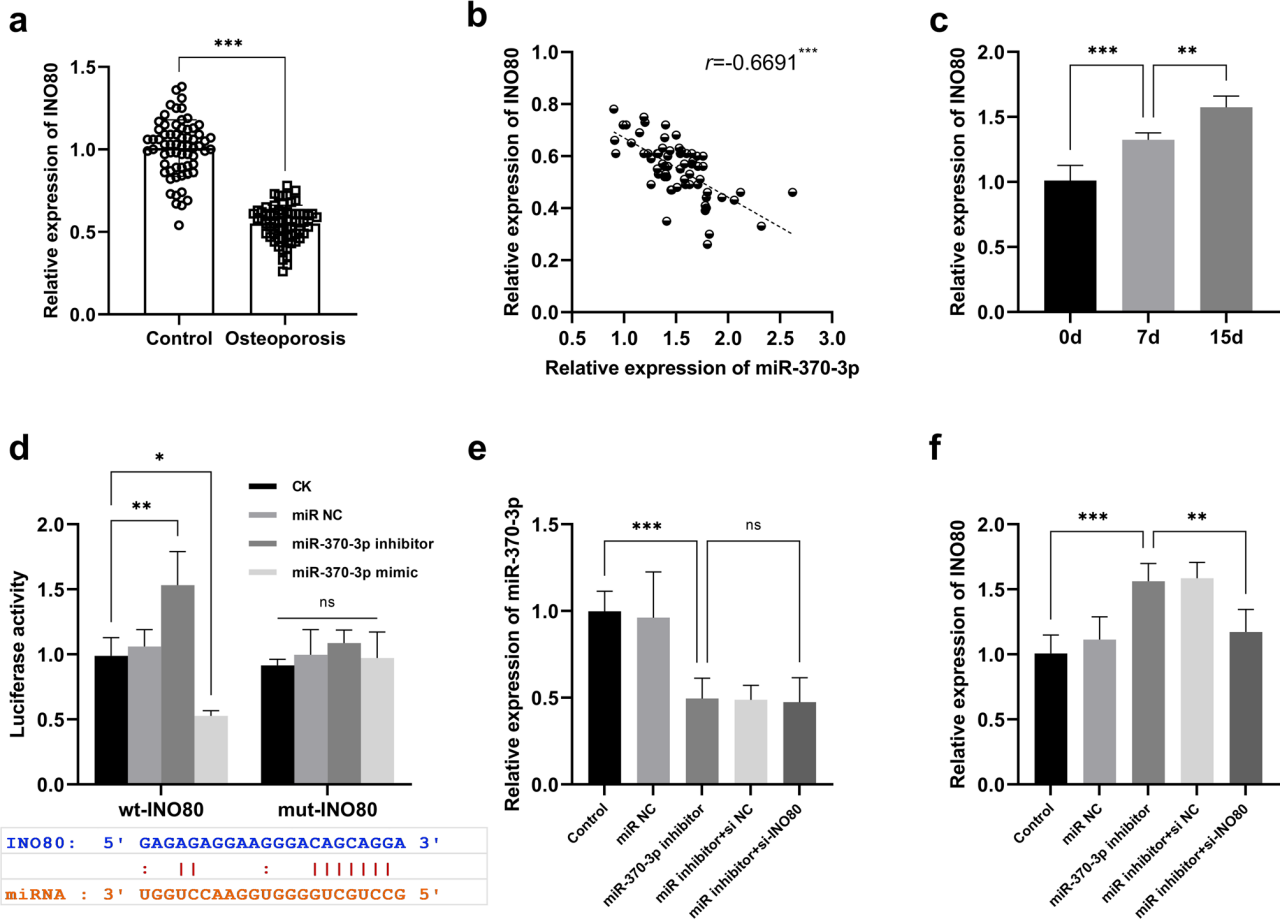
miR-370-3p could regulate the expression of INO80. ^***^p < 0.001, ^**^p < 0.01, ^*^p < 0.05. Expression of INO80 in control group (*n* = 65) and PMO patients (*n* = 64) (**a**). Correlation between miR-370-3p and INO80 expression (**b**). Expression of INO80 at different time of osteoblast differentiation (**c**). Dual-luciferase report gene assay was used to detect the targeting relationship between miR-370-3p and INO80 (**d**). The mutual regulatory relationship between miR-370-3p and INO80 (**e-f**)

The expression of INO80 was found to be downregulated in PMO patients (Fig. 4a), and a significant negative correlation was observed between INO80 and miR-370-3p levels in PMO (Fig. 4b). Furthermore, the expression of INO80 was shown to be upregulated as the duration of osteoblast differentiation increased (Fig. 4c). A dual-luciferase reporter gene assays further confirmed the direct interaction between INO80 and miR-370-3p (Fig. 4d). Moreover, miR-370-3p was demonstrated to regulate the expression of INO80 (Fig. 4e and f).

### Knockdown of INO80 partially mitigated the effects of miR-370-3p inhibitors on osteoblasts

**Fig. 5:**
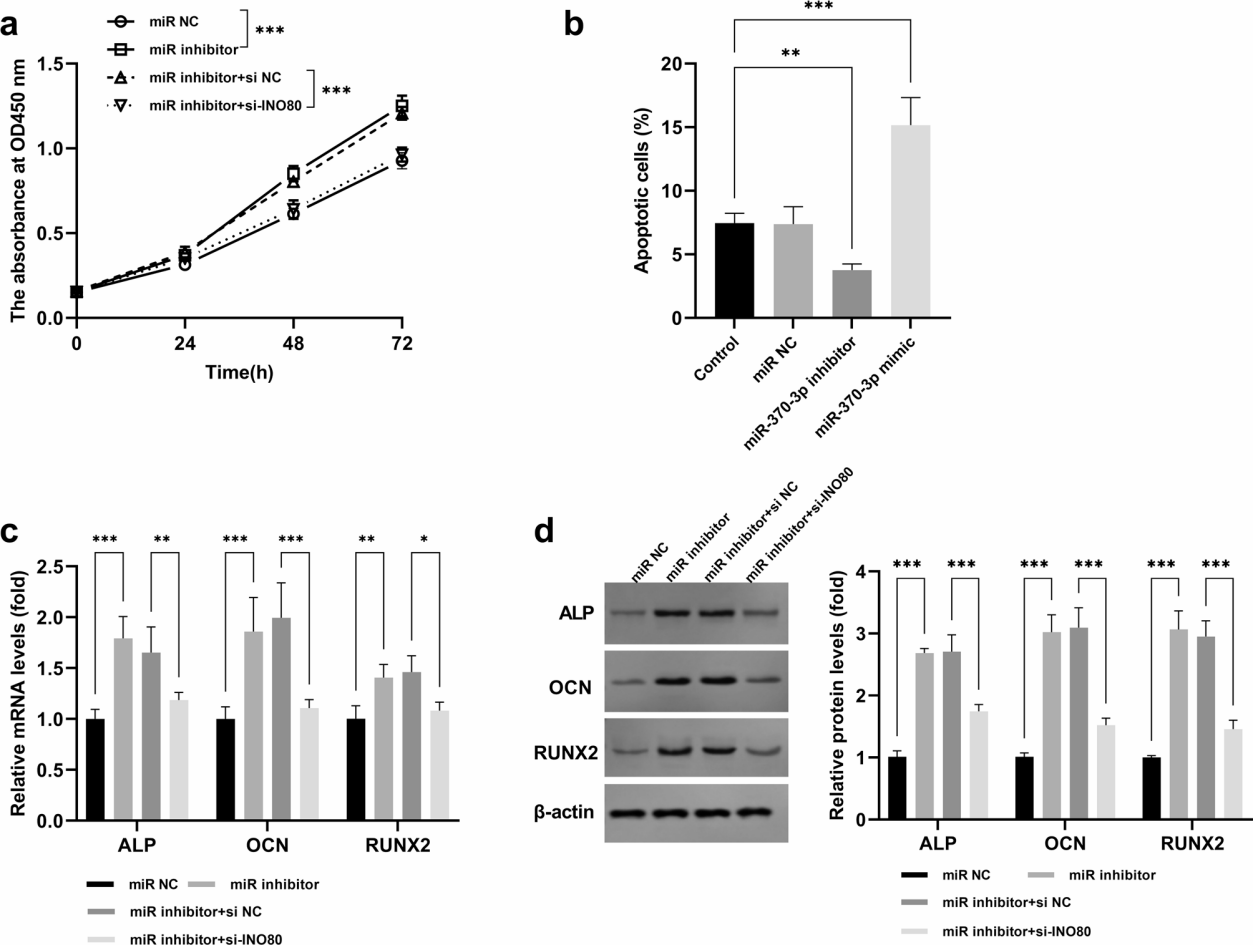
miR-370-3p affected the proliferation, apoptosis and differentiation of osteoblasts by regulating INO80. ^***^p < 0.001, ^**^p < 0.01, ^*^p < 0.05. Inhibition of INO80 partially reversed the effects of miR-370-3p on cell proliferation (**a**), apoptosis (**b**), mRNA (**c**) and protein (**d**) levels of osteoblast differentiation markers (**c-d**)

Knocking down INO80 reversed the effects of miR-370-3p inhibitor on osteoblast proliferation and apoptosis (Fig. 5a and b). Additionally, knocking down INO80 also counteracted the effect of miR-370-5p inhibitor on osteoblast differentiation. Specifically, compared with the sole inhibition of miR-370-3p, after knocking down INO80, the mRNA expression levels of ALP (0.66 ± 0.07-fold), OCN (0.60 ± 0.08-fold), and RUNX2 (0.77 ± 0.08-fold) decreased, and the protein expression levels of ALP (0.65 ± 0.11-fold), OCN (0.50 ± 0.11-fold), and RUNX2 (0.48 ± 0.14-fold) also decreased (Fig. 5c and d).

## Discussion

The challenge in the early detection of osteoporosis arises from the absence of distinct clinical manifestations during the initial stages [22]. Several biomarkers or therapeutic targets for OP have been identified, including bone transformation markers (BTM) [23, 24] and small interfering RNAs (siRNAs) [25]. miRNAs also play a role in regulating bone maintenance and contributing to the pathogenesis of osteoporosis [26]. Several miRNAs have been identified to modulate osteogenic activity and the formation of osteoblastic bone. For instance, miR-151a-3p has been found to inhibit osteoblasts viability, suggesting that it may represent a prospective therapeutic target for PMO [11]. Although previous studies have observed the abnormal expression of miR-370-3p in osteoporosis animal models, its clinical relevance in human PMO populations remains unclear; the functional connection between this molecule and the key bone metabolism regulator INO80 still lacks direct evidence at the mechanistic level. This study fills the gap in the clinical and molecular mechanisms of miR-370-3p in osteoporosis through a multi-dimensional verification system integrating clinical and cellular aspects.

We validated an increased expression of miR-370-3p in patients with PMO, findings that align closely with prior research [20], and investigated its potential clinical utility for diagnostic value. This suggested that miR-370-3p may be involved in the pathogenesis of PMO. Furthermore, we assessed key indices related to OP and conducted an analysis to determine the relationship between serum expression levels of miR-370-3p and BMD. The results revealed a negative association between miR-370-3p expression and BMD in PMO patients, providing further elucidation of the connection between miR-370-3p and OP in postmenopausal woman. Earlier research has indicated that several miRNAs can act as biomarkers for OP, including miR-497-5p [27], miR-100 [28], miR-32-3p [29], and others. Additionally, our study demonstrated that the combination of miR-370-3p and BMD significantly enhanced the diagnostic accuracy for OP in postmenopausal woman. Consequently, we inferred that a combination of miR-370-3p with other established miRNA biomarkers for OP could further improve the diagnostic performance, though additional data are required to substantiate this hypothesis.

The impairment of osteoblast function is a key initiating factor in the pathogenesis of OP. Consequently, investigating the precise roles of osteoblasts is essential for a deeper understanding of the molecular mechanisms underlying OP [30]. In addition, research has reported that miR-370-3p plays a significant role in the apoptosis of spinal cord neurons and trophoblast cells [31, 32]. Therefore, we speculate that miR-370-3p may affect osteoblast differentiation and apoptosis, thereby mediating the progression of osteoporosis. To verify this hypothesis, we designed a series of experiments to explore the specific effects of miR-370-3p on osteoblast behavior through overexpression or inhibition of miR-370-3p expression. The findings from these experiments further substantiated the validity of our study and provided additional insights into the regulatory mechanisms involved.

In fact, studies have also shown that the differentiation process of most osteoblasts is significantly affected by the regulation of multiple transcription factors and cytokines [33]. ALP is a marker of initial osteoblast maturation and serves as an indicator of bone matrix synthesis due to its upregulation as osteoblasts progress through the differentiation process [34]. OCN is a protein predominantly synthesized by osteoblasts and is the most prevalent non-collagenous fraction within bone tissue [35]. RUNX2 is predominantly expressed in osteoblasts and plays a crucial role in regulating the differentiation of these cells as well as the overall process of bone development [36]. Notably, ALP, RUNX2, and OPN have all been shown to significantly enhance osteogenic processes [37]. Our study revealed that miR-370-3p inhibition led to a marked increase in the expression levels of ALP, RUNX2, and OCN, while miR-370-3p mimic produced the opposite effect. Based on the above results and information, it can be speculated that miR-370-3p may affect the expression levels of ALP, RUNX2 and OCN by targeting certain negative regulatory factors or directly acting on the transcriptional network of osteogenic differentiation-related genes. However, inhibiting the expression of miR-370-3p may relieve this negative regulatory effect and promote the early differentiation of osteoblasts. This discovery not only provides a new perspective on the role of miR-370-3p in bone metabolism regulation, but also lays a theoretical foundation for the future development of treatment strategies for bone diseases such as osteoporosis.

The INO80 chromatin-remodeling complex plays a crucial role in regulating both transcriptional activation and repression [38]. Inhibition of the INO80 subunits leads to a reduction in mineral deposition in mesenchymal stem cells (MSC) under osteogenic conditions. Furthermore, suppression of INO80 expression in MSCs cultured under osteogenic conditions results in a significant decrease in the mRNA expression of osteoblast-specific genes [39]. To further explore the interaction between miR-370-3p and INO80, we designed a series of rescue experiments. Our study found that the expression level of INO80 is significantly reduced in PMO patients, which is a novel phenomenon and supplements the content not covered in the existing literature. This discovery suggests that INO80 is not only important for normal bone formation but also contributes to osteoporosis development. Our study has for the first time revealed the direct targeting regulatory mechanism of miR-370-3p on INO80. This regulatory relationship provided a reasonable mechanism basis for explaining the downregulation of INO80 in PMO patients. Further, our study found that downregulating INO80 counteracted miR-370-3p’s effects on osteoblast proliferation and apoptosis. This result indicates a complex dynamic balance between miR-370-3p and INO80, both of which jointly affect osteoblast function. This finding provides a potential direction for developing therapeutic strategies for osteoporosis. For example, regulating miR-370-3p or INO80 expression could restore osteoblast function and improve bone metabolism balance. Given the abnormally high expression of miR-370-3p in PMO patients, it is plausible that detecting miR-370-3p at the early stages of the disease could facilitate early diagnosis, enabling the timely initiation of therapeutic interventions that may positively impact the disease course. Moreover, it could inhibit the progression of OP by promoting INO80 protein expression, thereby promoting the proliferation and differentiation of osteoblasts.

Studies have found that miR-27a derived from mesenchymal stem cell-derived extracellular vesicles inhibits osteoporosis [40]. Exosomes are regarded as key mediators of intercellular communication because they carry many molecules, such as proteins, lipids and RNA [41]. Additionally, research has also reported that exosomes derived from senescent osteoblasts mediate miR-139-5p to regulate endothelial cell function [42]. In this study, the abnormal elevation of miR-370-3p in serum may result from the secretion of specific miRNA by osteoblasts under pathological conditions through exosomes or the paracrine effect of other bone microenvironment cells (such as mesenchymal stem cells). In osteoporosis, miRNA, as a molecular medium for intercellular communication, its changes in serum levels may reflect the pathological state of bone tissue, providing a theoretical basis for the early diagnosis of the disease. But this might require more clinical data for verification. Therefore, more experiments are needed in the future to explore the source of miR-370-3p in serum.

As an important biomarker, miRNA has shown great potential in the fields of disease diagnosis and treatment. For instance, in diagnosis, miRNA can provide clues for the early detection of diseases by detecting changes in expression levels in blood, saliva or other body fluids; while in the treatment field, by regulating the expression of specific mRNA [43], the disease process can be intervened, thus bringing new treatment hope to patients. However, to transform these potential applications into practical clinical solutions, several key challenges need to be overcome. Firstly, the stability and specificity of miRNA need to be further verified, especially in complex biological sample environments, ensuring the accuracy of detection results is a crucial issue. Secondly, the design of delivery systems is also a key link in realizing miRNA treatment [44].

The current study focuses on characterizing miR-370-3p in postmenopausal osteoporosis. However, the specific factors inducing its upregulation remain unclear, providing a key direction for future research. Recent studies have found that the expression of miR-370-3p may be closely related to the function of estrogen receptor α (ERα) [45]. As one of the main receptors of estrogen, ERα may regulate its transcriptional activity by directly binding to the promoter region of the miR-370-3p gene. In addition to osteoporosis, miR-370-3p exhibits a complex regulatory network in other diseases. For example, in spinal cord injury (SCI), lncRNA H19 acts as a sponge for miR-370-3p via the ceRNA mechanism [31]. Similarly, in sepsis, a ceRNA regulatory pattern was observed where lncRNA NEAT1 sponges miR-370-3p [46]. We speculate that miR-370-3p expression changes may occur via ceRNA competitive adsorption. Future research should explore this ceRNA mechanism to provide more evidence for its role in osteoporosis. Under osteogenic culture conditions, INO80-silenced mesenchymal stem cells (MSCs) showed reduced RUNX2 mRNA expression, INO80 also positively regulates canonical Wnt signaling [39]. In this study, silencing INO80 reduced RUNX2 expression, suggesting INO80 may directly or indirectly regulate RUNX2 and affect osteogenic differentiation. However, the precise relationship between INO80 and Wnt signaling requires further investigation.

Several limitations of this study should be acknowledged. For the definite clinical conclusion, the sample size of this study was relatively small. It is necessary to expand the sample size in subsequent studies and conduct independent verification by combining multi-center data. Hormone and vitamin D3 levels are closely related to osteoporosis, and the duration after menopause has a significant impact. Future research will further verify the relationship between estrogen, vitamin D3 and osteoporosis. Additionally, ALP staining and calcium nodule formation were not examined. Future research will include these steps to confirm osteoblast differentiation. Although we have conducted a preliminary exploration of miR-370-3p in osteoporosis and its impact on osteoblast differentiation, the specific mechanism of miR-370-3p’s role in the calcification process has not been further investigated. Calcification is one of the key steps in bone formation, and its regulatory mechanism is complex, involving multiple molecules and signaling pathways. Therefore, future research could consider systematically evaluating the role of miR-370-3p during the calcification stage of osteoblasts. In addition, animal experiments for verification will also be a key focus in future work. Future research plan to further explore the role of miR-370-3p/INO80 in postmenopausal osteoporosis by constructing an OVX animal model. Moreover, the expression specificity of miR-370-3p has not yet been confirmed. Future research should explore the specificity of miR-370-3p by including cross-skeletal disease cohorts (OA, osteoporosis and fractures). Finally, future research directions could further compare the efficacy of miR-370-3p with traditional markers (such as CTX-1, P1NP and BALP) in diagnosing osteoporosis, providing a more comprehensive reference basis for clinical application.

In general, this study explored the expression and clinical prediction of miR-370-3p in PMO, as well as to examine its role in modulating disease progression through the regulation of INO80 expression. Further investigations are planned to carry out additional experiments and gather more comprehensive data, thereby enhancing the significance and value of this study.

## Electronic supplementary material

Below is the link to the electronic supplementary material.

Supplementary Fig. 1. The expression level of miR-370-3p in an independent cohort

Supplementary Fig. 2. The mRNA levels of osteoblast differentiation markers at different time points

## Data Availability

The datasets used and/or analysed during the current study are available from the corresponding author on reasonable request.
