# Supplementary figures and images for: miR-370-3p affects the progression of postmenopausal osteoporosis through targeting INO80
Source: Hereditas. 2025 Jul 22;162:138. doi: 10.1186/s41065-025-00502-8 (PMC12285066; doi:10.1186/s41065-025-00502-8)

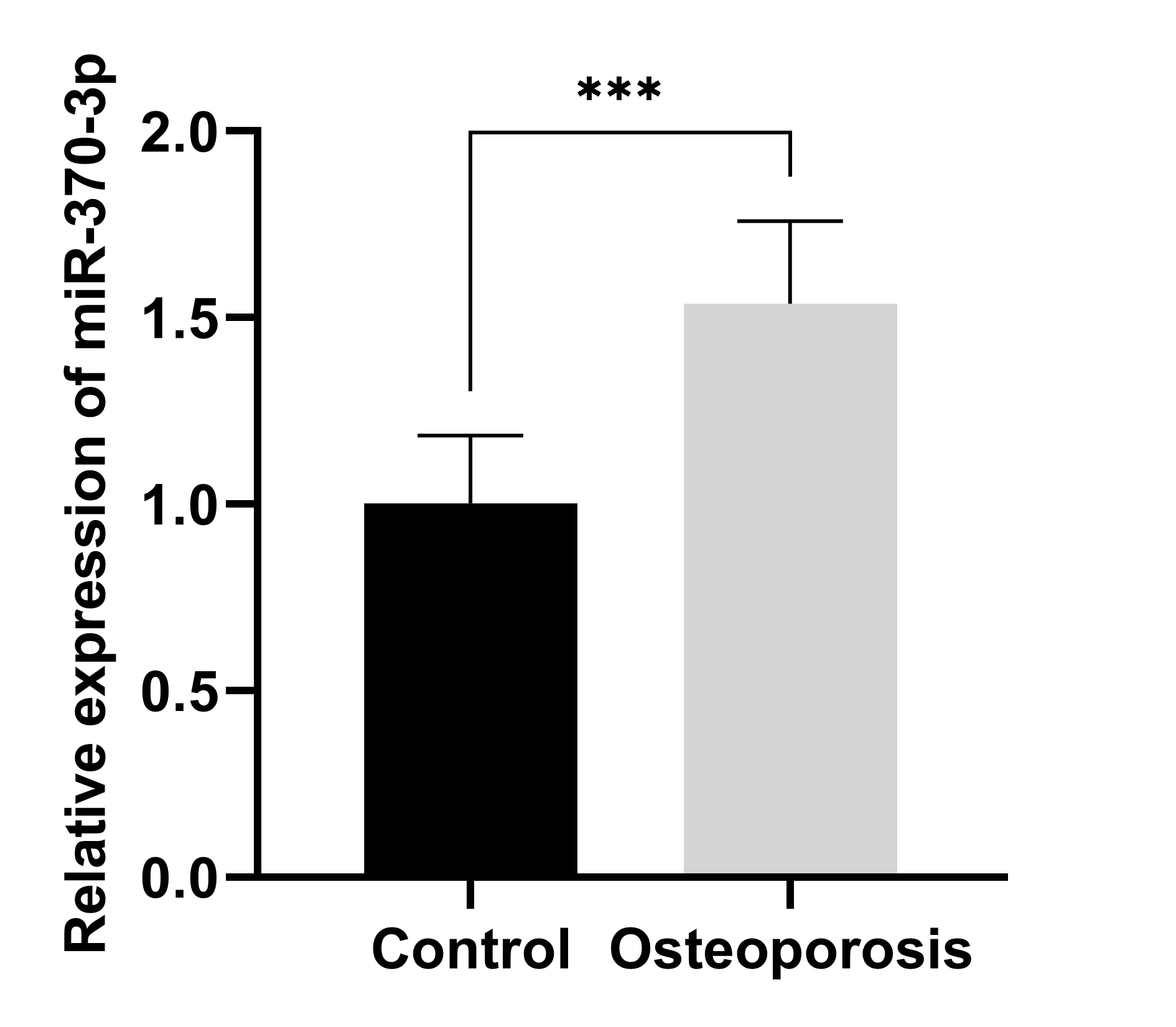

Supplement: Supplementary file 1 — Supplementary Fig. 1. The expression level of miR-370-3p in an independent cohort [file 41065_2025_502_MOESM1_ESM.tif]

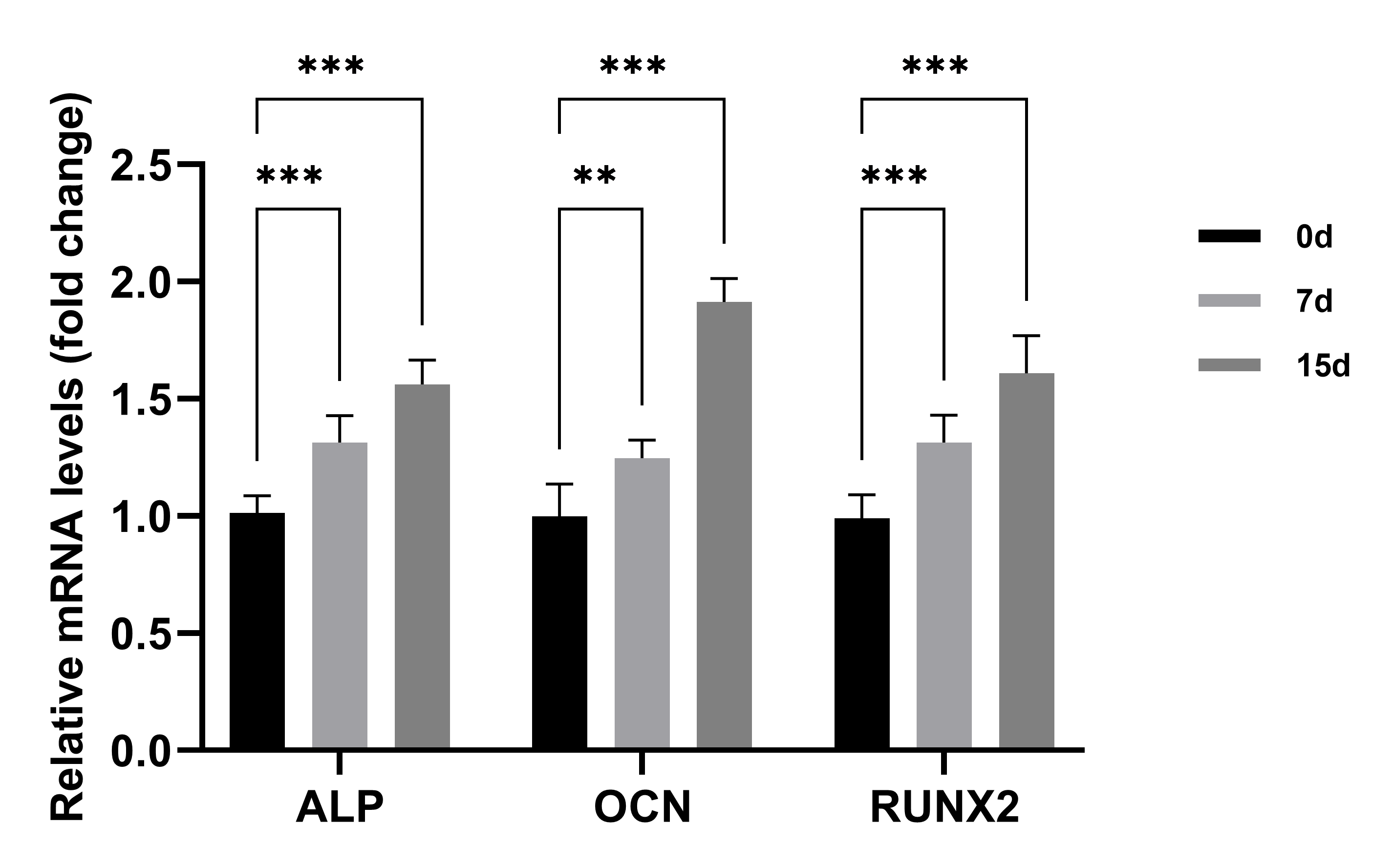

Supplement: Supplementary file 2 — Supplementary Fig. 2. The mRNA levels of osteoblast differentiation markers at different time points [file 41065_2025_502_MOESM2_ESM.tif]
